# A rapid review of home-based activities that can promote mental wellness during the COVID-19 pandemic

**DOI:** 10.1371/journal.pone.0243125

**Published:** 2020-12-03

**Authors:** Joseph H. Puyat, Haroon Ahmad, Ana Michelle Avina-Galindo, Arminee Kazanjian, Aanchel Gupta, Ursula Ellis, Maureen C. Ashe, Fidel Vila-Rodriguez, Priyanka Halli, Amy Salmon, Daniel Vigo, Alberto Almeida, Christopher E. De Bono

**Affiliations:** 1 Centre for Health Evaluation and Outcome Sciences, Providence Health Care and The University of British Columbia, Vancouver, Canada; 2 School of Population & Public Health, The University of British Columbia, Vancouver, Canada; 3 Department of Psychiatry, Faculty of Medicine, The University of British Columbia, Vancouver, Canada; 4 Non-Invasive Neurostimulation Therapies Laboratory, Department of Psychiatry, The University of British Columbia, Vancouver, Canada; 5 Woodward Library, The University of British Columbia, Vancouver, Canada; 6 Department of Family Practice, The University of British Columbia, Vancouver, Canada; 7 Mental Health Program, Providence Health Care, Vancouver, British Columbia, Canada; 8 Providence Health Care, Vancouver, British Columbia, Canada; National Cancer Center Japan, JAPAN

## Abstract

**Background:**

During the COVID-19 pandemic, public health measures such as isolation, quarantine, and social distancing are needed. Some of these measures can adversely affect mental health. Activities that can be performed at home may mitigate these consequences and improve overall mental well-being. In this study, home-based activities that have potential beneficial effects on mental health were examined.

**Methods:**

A rapid review was conducted based on a search of the following databases: MEDLINE, EMBASE, CINAHL, PyscINFO, Global Health, epistemonikos.org, covid19reviews.org, and eppi.ioe.ac.uk/covid19_map_v13.html. Eligible studies include randomized controlled trials and non-randomized studies published between 1/1/2000 and 28/05/2020 and that examined the impact of various activities on mental health outcomes in low-resource settings and contexts that lead to social isolation. Studies of activities that require mental health professionals or that could not be done at home were excluded. Two review authors performed title/abstract screening. At the full-text review stage, 25% of the potentially eligible studies were reviewed in full by two review authors; the rest were reviewed by one review author. Risk of bias assessment and data extraction were performed by one review author and checked by a second review author. The main outcome assessed was change or differences in mental health as expressed in Cohen’s d; analysis was conducted following the synthesis without meta-analysis guidelines (SWiM). PROSPERO registration: CRD42020186082.

**Results:**

Of 1,236 unique records identified, 160 were reviewed in full, resulting in 16 included studies. The included studies reported on the beneficial effects of exercise, yoga, progressive muscle relaxation, and listening to relaxing music. One study reported on the association between solitary religious activities and post traumatic stress disorder symptoms. While most of the included studies examined activities in group settings, particularly among individuals in prisons, the activities were described as something that can be performed at home and alone. All included studies were assessed to be at risk of bias in one or more of the bias domains examined.

**Conclusions:**

There is some evidence that certain home-based activities can promote mental wellness during the COVID-19 pandemic. Guidelines are needed to help optimize benefits while minimizing potential risks when performing these activities.

## Background

Without vaccines and effective treatments, public health approaches to controlling the COVID-19 pandemic will continue to depend on measures such as isolation, quarantine, physical distancing, and increased hand-washing. Some of these measures, such as physical distancing and quarantine, may negatively impact mental health when implemented simultaneously and for long periods of time [1]. This in turn can influence adherence to the public health measures [2], potentially undermining their effectiveness over time.

Evidence-based strategies are urgently needed to mitigate the short- and long-term impact that some public health measures might have on mental health. As access to in-person health resources may be limited during the COVID-19 public health emergency, alternative and complementary activities that promote mental health and wellbeing need to be explored and identified. Of particular importance are activities that can be performed at home and that do not require facilitation by mental health professionals. These activities include those that can be performed by an individual or in groups as facilitated by video conferencing platforms.

Currently, there is extensive literature on the impact of various activities on mental health in general. To our knowledge, there has been no study that has examined activities that can promote mental wellness during pandemics, quarantines, social isolation or other stress-inducing events. This study was conducted to help address that gap in knowledge.

## Methods

This rapid review study was informed by the guidelines provided by the Cochrane Collaboration [3].

### Criteria for eligible studies

Studies considered in this review include randomized clinical trials and non-randomized studies such as cohort studies, case-control studies, and cross-sectional studies. No restrictions were placed on study duration, participants, setting (group-based or individual-based) or comparators.

To be considered for full review, studies must contain results that examine the impact on mental health of various activities that can be performed at home. Studies must also have been conducted in specific contexts that lead to social isolation, such as pandemics, post-disaster periods, incarceration, and scientific expeditions or explorations. We did not include studies that focused on a different type of chronic social isolation that can develop over long periods of time and that disproportionately affects many older people or those with chronic health conditions. It has been argued that this type of social isolation differs from the massive, abrupt, and extreme nature of social isolation that happens during pandemic [4]. Currently, it is not known how much of the literature on chronic social isolation can be applied to the COVID-19 pandemic or future pandemics.

An additional criterion for inclusion is that studies must have used standardized mental health outcome measures to examine group differences or changes over time, such as depression, anxiety, post-traumatic stress disorder, and psychological distress.

We excluded studies that were not in English, published before 2000, or that examined interventions or activities that can only be done at a hospital or with the active support or facilitation of a therapist or a mental health professional, such as group psychotherapy, for example. Studies that were done in group settings and that involve instructors, like yoga or fitness instructors, were not excluded if the activities can also be performed on an individual-basis with the aid of video or live streamed instructional materials.

As a final exclusion criterion, we restricted our search to studies published in the last two decades to limit the scope to studies that are most likely to be relevant to recent pandemics.

### Search strategy

The search for eligible studies was performed following procedures described in the rapid review protocol registered in PROSPERO on May 20, 2020 (#CRD42020186082) [5]. A search strategy was developed with the assistance of an information specialist (UE) and reviewed by a librarian who is not a member of the research team.

The search was performed on May 28, 2020 by an information specialist (UE) using five bibliographic databases (Ovid MEDLINE, Ovid EMBASE, CINAHL, PsycINFO, CAB Global Health) and three other sources (epistemonikos.org, covid19reviews.org, eppi.ioe.ac.uk/covid19_map_v13.html). Please see S1 Appendix for the complete Ovid MEDLINE search strategy. A supplementary search for ongoing relevant trials was also performed (AG) on July 10, 2020 in the Australian New Zealand Clinical Trials Registry, ClinicalTrials.gov, Chinese Clinical Trial Registry, and German Clinical Trials Registry.

### Screening

Title and abstract screening were independently conducted by two review authors (AMA, JHP, or HA). For 25% of the potentially eligible records, full review was conducted by two review authors (AMA and JHP); the rest were reviewed by one review author (AMA) with a second review author (JHP or HA) conducting full reviews on all excluded studies. We resolved all conflicts by consensus and we recorded all reasons for excluding studies during the full-text review. We used the Covidence [6] platform for screening and full-text review.

### Data collection and analysis

Data extraction was performed in Excel by one review author (AMA or HA) and verified by another review author (JHP or AG). For each of the included studies, we extracted data on study characteristics (year, authors, title, journal, country), study design (methods, study setting, number of participants), participant characteristics (age, sex, mental health status), intervention characteristics (type of intervention, duration), mental health outcomes assessed, and effect measures (means and standard deviations, change in proportions, regression coefficients and standard errors, and correlation coefficients). Data extraction was restricted to only what was reported in the published studies.

To assess eligible RCTs for risk of bias, we used the ‘Risk of bias’ (RoB 2.0) tool [7]. For eligible pre-post and cross-sectional studies, we used the risk of bias tool to assess non-randomized studies of interventions (ROBINS-I) [8]. Studies were considered to be at high risk of bias overall if they were assessed to be “high” risk in any of the five RoB domains or if they have “serious” or “critical” risk of bias in one or more of the seven ROBINS-I domains. All assessments of risk of bias were performed by one review author (JHP). Results from the risk of bias assessments were summarized with the aid of the robvis visualization tool [9].

As prespecified in the registered protocol [5], no meta-analysis was performed due to the variety and heterogeneity of the studies. Nevertheless, we used the Synthesis without meta-analysis (SWiM) in systematic reviews: reporting guideline [10] to organize and present our review findings. For example, we used tables to group eligible studies according to type of activities that suggest common underlying mechanisms for improving mental wellness. We converted all measures of treatment into effect size estimates (Cohen’s d) with corresponding 95% confidence intervals to facilitate the description of treatment effects across studies that use different outcome and treatment effect measures. These effect size estimates were sorted by magnitude and presented as forest plots, without pooled results. If the body of evidence allowed, we were also prepared to conduct subgroup analysis to assess whether interventions were helpful for specific subgroups such as persons with mental illness, persons at risk of developing symptoms, essential services workers, or persons from different socio-demographic groups like adolescents, older adults, women and men.

## Results

**Fig 1:**
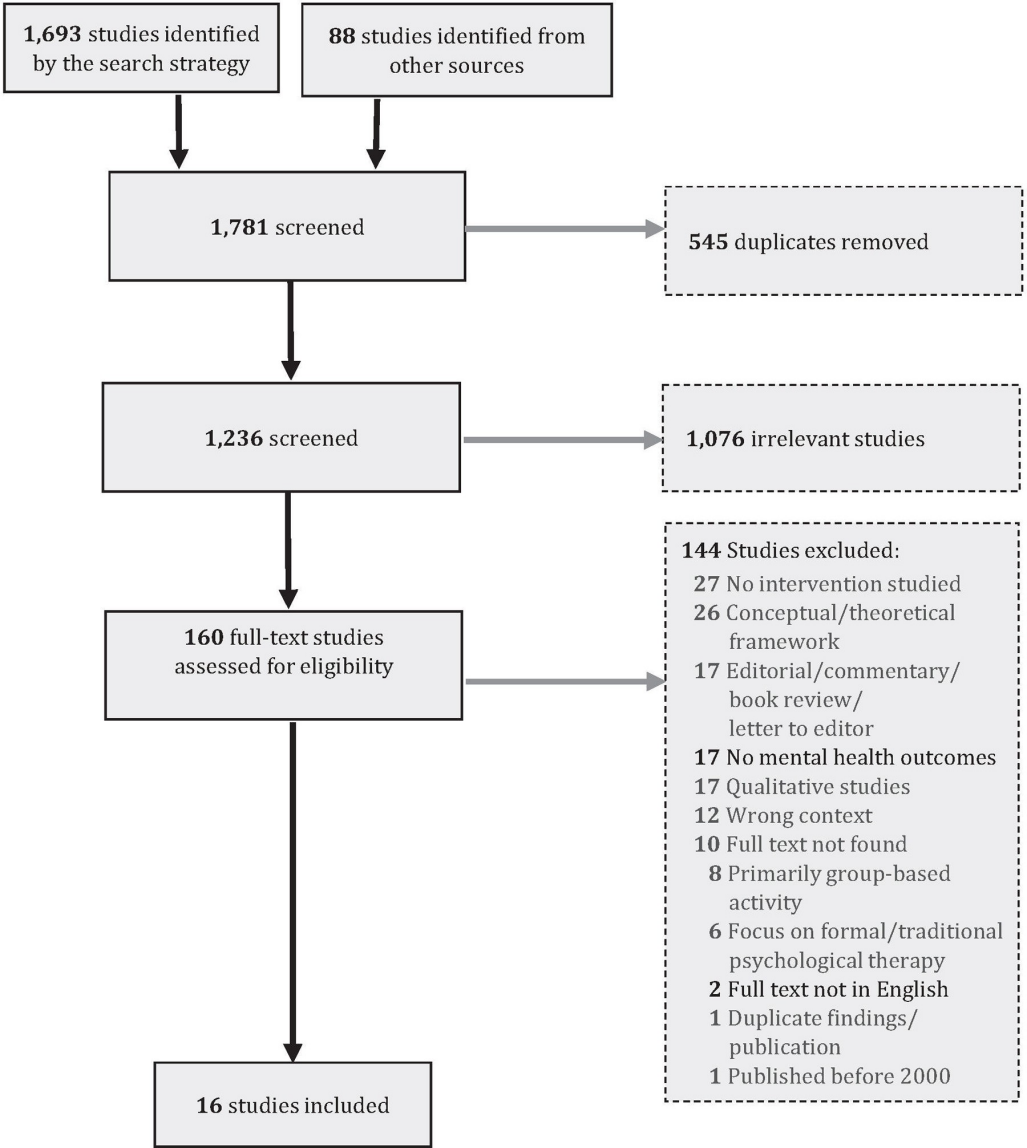
Study flow diagram.

Our search identified a total of 1,781 records obtained from databases and from backward and forward searches. After removing all duplicates, we screened 1,236 unique titles and abstracts and excluded 1,076. Of the 160 articles that were reviewed in full, 16 met all study criteria and 144 were excluded. The reasons for excluding the articles and the review process are summarized in Fig 1.

The 16 included studies, half of which were RCTs, investigated the effects of five types of activities on various mental health outcomes. The most frequently investigated activities were exercise (eight studies) and yoga (five studies). The mental health outcomes examined pertain to reduction in anxiety, depression, psychological distress, feelings of hopelessness, and improvement in overall symptom severity.

Of the 16 included studies, 13 were conducted in prisons; the other 3 were conducted in a forensic psychiatry unit, hospital ward, and community setting. All of the exercise studies were conducted in group settings with no indications that the activity could not be performed alone. Likewise, in the yoga studies, which were all done in classes led by a yoga instructor, the activity was described as an activity that could be performed alone. Only one study, an RCT investigating the effect of progressive muscle relaxation technique on anxiety in a sample of hospitalized individuals, was conducted during the COVID-19 pandemic.

A summary of the characteristics of each included study and details on the reported outcomes are presented on Table 1.

**Table 1:** Summary of study characteristics.

Study ID	Country	Design	Participants	Setting	Intervention/Activities	Mental Health Outcome	Results
**EXERCISE**							
Legrand et al (2020) [11]	France	RCT	37 first-time prisoners with elevated anxiety	Prison	6-week interval exercise program (40 minutes/day)	Anxiety measured via State-Trait Anxiety Inventory	Exercise group (n = 20) Wait-list group (n = 17) Average reduction in state anxiety scores in the exercise group (-6.6, SD = 7.5) was greater than the average reduction in the wait-list group (-1.5, SD = 7.2). *d* = 0.69 (95%CI: .02, 1.35)
Psychou et al (2020) [12]	Greece	RCT	60 prisoners	Prison	12-week exercise program, 3 sessions per week, 60 minutes in duration	Mood states measured via Profile of Mood States (POMS) and Anxiety via The State-Trait Anxiety Inventory (STAI)	Exercise group (n = 35) Control group (n = 25) The intervention group when compared with the control group had lower mean scores on the following outcomes: Depression (1.42, SD = .63 vs 2.65, SD = .78) Total mood profile (103.24, SD = 32.3 vs 109.99, SD = 3.73) State anxiety (1.70, SD = .31 vs 2.92, SD = .53) Trait anxiety (1.96, SD = .26 vs 2.65, SD = .42) *d*: Depression = 1.77 (95%CI: 1.15, 2.37) State anxiety = 2.94 (95%CI: 2.19, 3.67) Trait anxiety = 2.06 (95%CI: 1.42, 2.68)
O’Toole et al (2018) [13]	Ireland	Pre-post	30 prisoners pre-existing mental health symptoms	Prison	3-month exercise program	Depression, anxiety and stress(DASS-42), Novaco Anger Scale, Rosenberg Self-Esteem Scale, Zung Self-Rated Anxiety Scale	N = 30 Post-intervention, there was a decrease in the proportion of participants with moderate to extreme scores in the following outcomes: Depression: (53.3% vs 10%) *d* = 0.99 (95%CI: 0.22, 1.75) Anxiety: (63.3% vs 20%) *d* = 0.91 (95%CI: 0.15, 1.66)
Zar et al (2017) [14]	Iran	Cross-sectional	97 prisoners	Prison	At least 3 exercise sessions each week	Anxiety, social Dysfunction, Depression measured via Goldberg & Hild's General Health Questionnaire	Exercise group (n = 44) Control group (n = 53) The intervention group when compared with the control group had lower mean scores on the following outcomes: Somatization (8.02, SD = 4.64 vs 11.16, SD = 5.52) Anxiety (7.90, SD = 4.60 vs 11.07, SD = 5.45) Social dysfunction (7.32, SD = 4.25 vs 8.66, SD = 5.43) Severe depression (6.55, SD = 5.15 vs 10.96, SD = 6.10) *d*: Somatization = 0.61 (95%CI: 0.20, 1.02) Anxiety = 0.62 (95%CI: 0.21, 1.03) Social dysfunction = 0.27 (95%CI: -0.13, 0.67) Severe depression = 0.78 (95%CI: 0.36, 1.19)
Baidawi et al (2016) [15]	Australia	Cross-sectional	173 prisoners, predominantly first-time inmates	Prison	Exercise over 4 weeks	Psychological distress measured via Kessler Psychological Distress Scale—K10	Participants who engaged in exercise in the previous month compared with those who did not exercise had, on average, 0.89 (95% CI: .25, 1.53) lower distress scores, after adjusting for potential confounders. *d* = 0.16 (95%CI: -0.19, 0.51)
Battaglia et al (2015) [16]	Italy	RCT	64 prisoners	Prison	9 months of cardiovascular plus resistance training (CRT); or high-intensity strength training (HIST)	Mental disorder symptoms using the Symptom Checklist-90-Revised (SCL-90-R)	Exercise group CRT (n = 22) Exercise group HIST (n = 22) Control group CRT (n = 20) The CRT intervention group when compared with the control group had lower mean scores on: Somatization (0.20, SD = 0.27 vs 0.23, SD = 0.24) Obsessive-compulsive (0.36, SD = 0.59 vs 0.27, SD = 0.25) Interpersonal Sensitivity (0.24, SD = 0.66 vs 0.22, SD = 0.35) Depression (0.26, SD = 0.40 vs 0.77, SD = 0.87) Anxiety (0.22, SD = 0.40 vs 0.28, SD = 0.50) Phobic anxiety (0.13, SD = 0.27 vs 0.11, SD = 0.19) Psychoticism (0.31, SD = 0.53 vs 0.24, SD = 0.29) Paranoid ideation (0.28, SD = 0.51 vs 0.43, SD = 0.46) GSI (0.26, SD = 0.40 vs 0.34, SD = 0.21) *d* (CRT): Somatization = 0.12 (95%CI: -0.49, 0.72) Obsessive-compulsive = 0.20 (95%CI: -0.41, 0.80) Interpersonal Sensitivity = 0.04 (95%CI: -0.57, 0.64) Depression = 0.77 (95%CI: 0.13, 1.39) Anxiety = 0.13 (95%CI: -0.47, 0.74) Phobic anxiety = 0.08 (95%CI: -0.52, 0.69) Psychoticism = 0.16 (95%CI: -0.45, 0.77 Paranoid ideation = 0.31 (95%CI: -0.30, 0.92) GSI = 0.25 (95%CI: -0.36, 0.85) The HIST intervention group when compared with the control group had lower mean scores on: Somatization (0.34, SD = 0.43 vs 0.23, SD = 0.24) Obsessive-compulsive (0.49, SD = 0.63 vs 0.27, SD = 0.25) Interpersonal Sensitivity (0.23, SD = 0.40 vs 0.22, SD = 0.35) Depression (0.24, SD = 0.27 vs 0.77, SD = 0.87) Anxiety (0.25, SD = 0.29 vs 0.28, SD = 0.50) Phobic anxiety (0.03 SD = 0.08 vs 0.11, SD = 0.19) Psychoticism (0.30, SD = 0.28 vs 0.24, SD = 0.29) Paranoid ideation (0.43, SD = 0.51 vs 0.43, SD = 0.46 GSI (0.32, SD = 0.33 vs 0.34, SD = 0.21) *d* (HIST): Somatization = 0.31 (95%CI: -0.30, 0.92) Obsessive-compulsive = 0.45 (95%CI: -0.17, 1.06) Interpersonal Sensitivity = 0.03 (95%CI: -0.58, 0.63) Depression = 0.84 (95%CI: 0.20, 1.47) Anxiety = 0.07 (95%CI: -0.53, 0.68) Phobic anxiety = 0.56 (95%CI: -0.06, 1.17) Psychoticism = 0.21 (95%CI: -0.40, 0.82) Paranoid ideation = 0.00 (95%CI: -0.61, 0.61) GSI = 0.07 (95%CI: -0.53, 0.68)
Buckaloo et al (2009) [17]	US	Cross-sectional	60 male prisoners	Prison	Exercise	Depression via Beck Depression Inventory and stress via Daily Hassles Scale	Exercise group (n = 30) No exercise group (n = 30) Mean depression scores in the exercise group (12.9, SD = 12.2) was lower compared to the no exercise group (22.5, SD = 8.8). *d* = 0.90 (95%CI = .37, 1.43) Mean scores on the DHS in the exercise group (52.6, SD = 49.0) was lower compared to the no exercise group (78.5, SD = 46.8). *d* = .56 (95%CI: -0.06, 1.17)
Cashin et al (2008) [18]	Australia	Cross-sectional	838 prisoners	Prison	Exercise over 4 weeks	Psychological well-being as measured by Beck Hopelessness Scale	Exercise was correlated with lower scores on BHS (r = -0.079, p <0.02). *d* = .16 (95%CI: 0.02, 0.29)
**YOGA (5 studies)**							
Sfendla et al (2018) [19]	Sweden	RCT	152 prisoners	Prison	10-week yoga, 90-min each week	Psychological distress measured via Brief Symptom Inventory (BSI)	Yoga group (n = 77) Control group (n = 75) The intervention group when compared with the control group had lower mean scores on: GSI (0.6, SD = 0.6 vs 0.8, SD = 0.7) Anxiety (0.8, SD = 0.8 vs 0.9, SD = 0.9) Depression (0.7, SD = 0.7 vs 1.0, SD = 1.0) Interpersonal Sensitivity (0.4, SD = 0.6 vs 0.8, SD = 1.0) Obsessive-compulsive (0.8, SD = 0.7 vs 1.2, SD = 0.9) Psychoticism (0.5, SD = 0.7 vs 0.7, SD = 0.8) Paranoid ideation (0.7, SD = 0.7 vs 1.0, SD = 0.9) Phobic anxiety (0.3, SD = 0.7 vs 0.5, SD = 0.9) Somatization (0.4, SD = 0.6 vs 0.7, SD = 0.6) *d*: GSI = 0.31 (95%CI: -0.01, 0.63) Anxiety = 0.12 (95%CI: -0.20, 0.44) Depression = 0.35 (95%CI: 0.03, 0.67) Interpersonal Sensitivity = 0.49 (95%CI: 0.16, 0.81) Obsessive-compulsive = 0.50 (95%CI: 0.17, 0.81) Psychoticism = 0.27 (95%CI: -0.05, 0.59) Paranoid ideation = 0.37 (95%CI: 0.05, 0.69) Phobic anxiety = 0.25 (95%CI: -0.07, 0.57) Somatization = 0.50 (95%CI: 0.18, 0.82)
Kerekes et al (2017) [20]	Sweden	RCT	152 prisoners	Prison	10-week yoga, 90-min each week	Perceived stress measured via PSS and psychological distress measured via BSI	Yoga group (n = 77) Wait-list group (n = 75) The intervention group when compared with the wait-list group had lower mean scores on the following outcomes: Perceived stress (22.4, SD = 9.0 vs 25.7, SD = 9.3) Psychological distress (0.6, SD = 0.6 vs 0.8, SD = 0.7) *d*: Perceived stress = 0.35 (95%CI: 0.03, 0.67) Psychological distress = 0.31 (95%CI: -0.01, 0.63)
Sistig et al (2015) [21]	New Zealand	Pre-Post	26 forensic patients	Forensic Psychiatry Centre	8-week mindful yoga sessions, weekly, 60-min in duration	Anxiety and Depression measured via Hospital Anxiety and Depression Scale (HADS), PSS, The Clinical Outcomes in Routine Evaluation–Outcome Measure (CORE- OM)	Anxiety scores decreased from 7.92 (SD = 4.39), at baseline, to 7.66 (SD = 4.03), post-intervention, and to 6.67 (SD = 3.83), 2 months post-intervention. *d* = 0.11 (95%CI: -0.66, 0.87) *d* = 0.58 (95%CI: -0.21, 1.36) Depression scores increased slightly from 5.12 (SD = 3.00) to 5.23 (SD = 3.92), post-intervention, but decreased to 4.58(SD = 3.35), 2 months post-intervention. *d* = -0.07 (95%CI: -0.84, 0.70) *d* = 0.31 (95%CI: -0.47, 1.08) Scores on perceived stress decreased slightly from 16.80 (SD = 7.98) to 16.46 (SD = 7.89), but increased to 17.51 (SD = 7.13), 2 months post-intervention. *d* = 0.09 (95%CI: -0.68, 0.86) *d* = -0.22 (95%CI: -0.98, 0.56)
Bilderbeck et al (2013) [22]	United Kingdom	RCT	100 prisoners	Prison	10-week yoga, weekly, 2 hours in duration	Perceived Stress measured via PSS, psychological distress measured via BSI, and positive and negative state affect measured via PANAS	Yoga group (n = 45) Control group (n = 55) The intervention group when compared with the control group had lower mean scores on the following outcomes: Positive affect (37.16, SD = 1.16 vs 31.22, SD = 1.02) Negative affect (15.02, SD = 0.80 vs 19.15, SD = 1.10) Perceived stress (11.40, SD = 1.10 vs 16.07, SD = 1.05) Psychological distress (24.49, SD = 3.38 vs 37.09, SD = 3.97) *d*: Positive affect = 5.47 (95%CI: 4.61, 6.33) Negative affect = 4.23 (95%CI: 3.51, 4.93) Perceived stress = 4.35 (95%CI: 3.62, 5.07) Psychological distress = 3.39 (95%CI: 2.77, 4.00)
Harner et al (2010) [23]	US	Pre-post	21 women prisoners	Prison	12-week yoga, 2 times per week, duration of 120 min	Depression, anxiety and stress as measured by Beck Depression Inventory, Beck Anxiety Inventory, Perceived Stress Scale (PSS)	At 12 weeks, depression scores decreased to 5.67 (SD = 7.5) from 24.90 (SD = 13.3) at baseline. *d* = 1.79 (95%CI: 1.07, 2.51) At 12 weeks, anxiety scores decreased to 7.3 (SD = 6.3) from 12.0 (SD = 13.8) at baseline. *d* = 0.44 (95%CI: -0.18, 1.05) Perceived stress scores were similar at 12 weeks, 22.0 (SD = 6.2) versus .22.8 (SD = 3.5) at baseline, *d* = 0.15 (95%CI: -0.46, 0.76)
**OTHERS (3 studies)**							
Liu et al (2020) [24]	China	RCT	51 COVID-19 patients	Hospital	5-day Progressive Muscle Relaxation (30 minutes/day)	Anxiety measured via State-Trait Anxiety Inventory	Intervention group (n = 25) Control group (n = 26) Mean state anxiety scores in the intervention group was lower (44.96, SD = 12.68) than the control group (57.15, SD = 9.24) *d* = 1.10 (95%CI: 0.51, 1.69)
Bensimon et al (2015) [25]	Israel	RCT	48 prisoners	Prison	3 weeks of listening to relaxing music; 45 minutes in duration, 3 times a day	Anxiety measured via State-Trait Anxiety Inventory (STAI), State-Trait Anger Expression Inventory (STAXI)	Music Group (n = 24) Control Group (n = 24) At week 3 of the intervention, the music group reported greater reduction (from baseline) in state anxiety than the control group (.27, 95%CI: 0.12, 0.42). *d* = 3.53 (95%CI: 2.61, 4.43) 1 week post-intervention, the greater reduction (from baseline) in state anxiety in the music group was sustained (.11, 95%CI: -0.04, 0.26). *d* = 1.44 (95%CI: 0.79, 2.07)
Cherry et al (2015) [26]	US	Cross-sectional	219 coastal residents	Community; post-disaster	Non-organizational religiosity	PTSD (17-item PTSD checklist); Depression (PHQ-9); and anxiety (GAD-7)	Increased odds of experiencing PTSD were reported among persons who were categorized as being medium, 2.03 (95% CI: 0.37, 11.3), and high, 9.8 (95%CI: 1.78, 53.78), in non-organizational religiosity when compared with persons who are low on non-organizational. d*medium* = -0.39 (95%CI: -1.34, 0.55) d*high* = -1.26 (95%CI: -2.20, -0.32)

*Abbreviations: BAI = Beck Anxiety Inventory; BDI = Beck Depression Inventory; BHS = Beck Hopelessness Scale; BSI = Brief Symptom Inventory; BSI-11 = Barratt Impulsiveness Scale; CORE-OM = Clinical Outcomes in Routine Evaluation- Outcome Measure; d = Cohen’s d or effect size; DASS-42 = Depression Anxiety Stress Scale; GAD-7 = General Anxiety Disorder-7; GHQ = General Health Questionnaire; HADS = Hospital Anxiety and Depression Scale; K10 = Kessler Psychological Distress Scale; NAS = Novaco Anger Scale; PANAS = Positive and Negative Affect Schedule; PCL = Posttraumatic Stress Disorder Checklist; PHQ-9 = Patient Health Questionnaire-9; POMS = Profile of Mood States; PSS = Perceived Stress Scale; RSES = Rosenberg Self-Esteem Scale; SAS = Zung Self-Rated Anxiety Scale; SCL-90-R = Symptom Checklist-90-Revised; STAI = State Trait Anxiety Inventory; STAXI = State-Trait Anger Expression Inventory.

### Risk of bias in included studies

**Fig 2:**
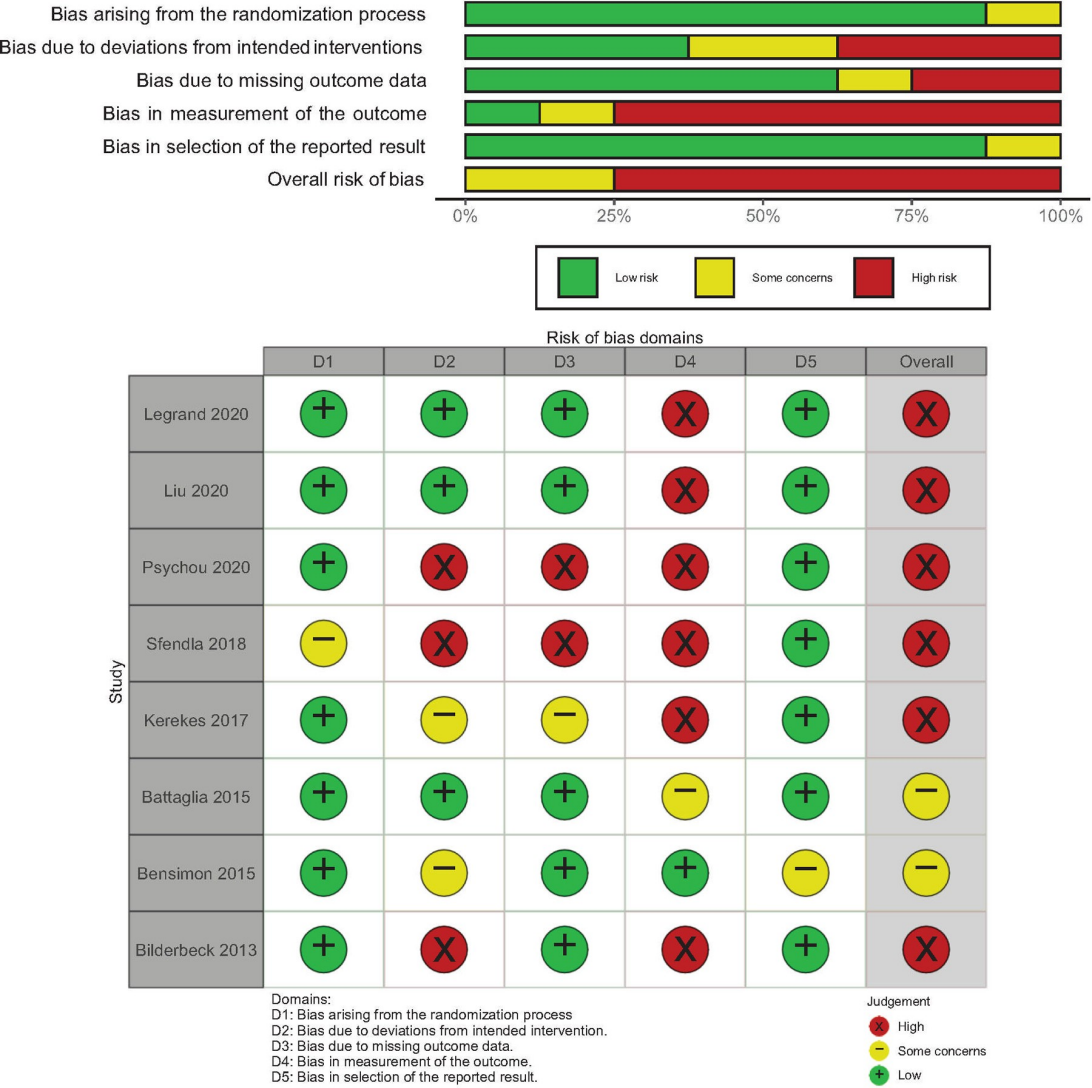
Risk of bias assessment for the included RCT.

**Fig 3:**
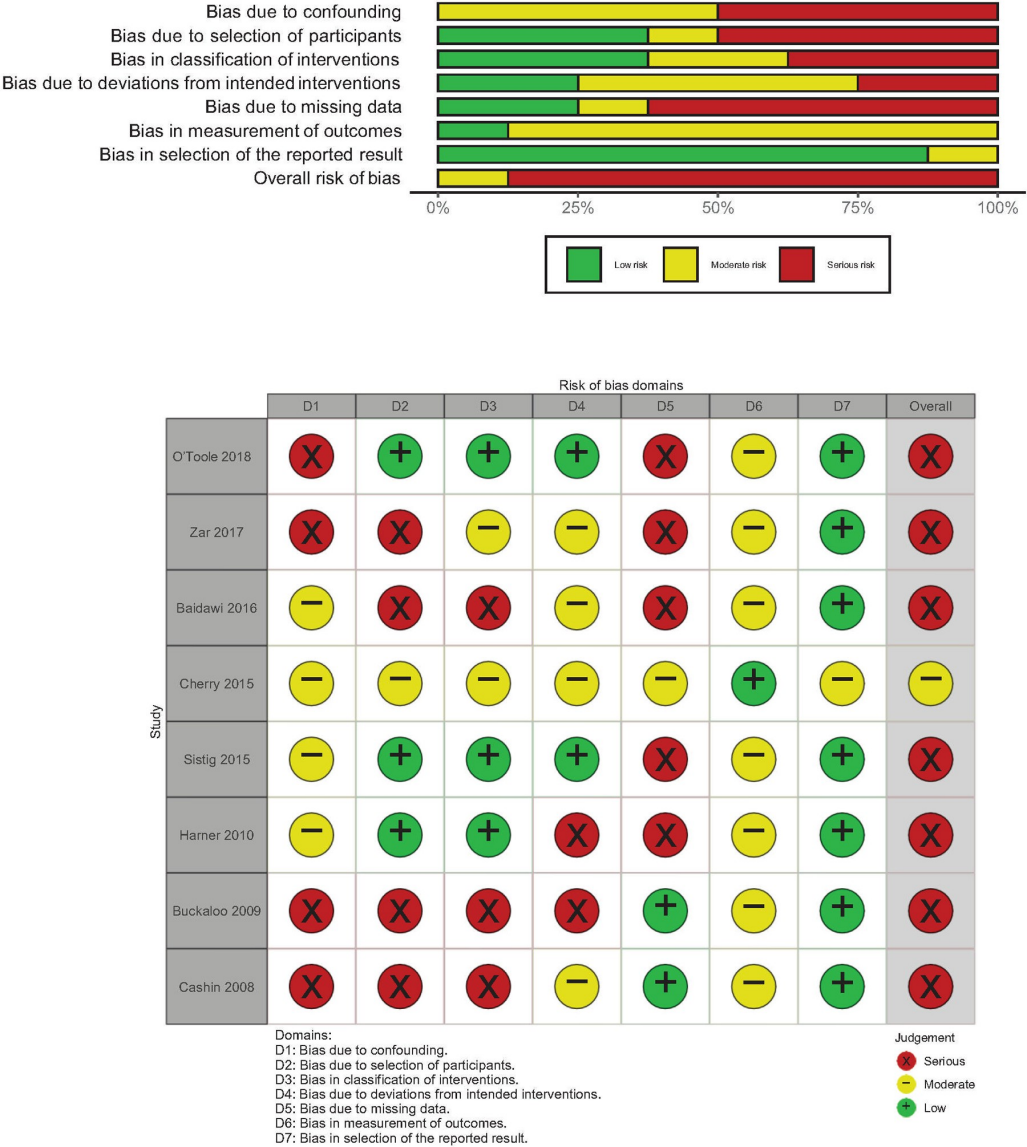
Risk of bias assessment for the included non-randomized studies of intervention.

The risk of bias assessments for the eight RCTs are summarized in Fig 2. Many of the studies were assessed to be at risk of bias or have some concerns in one of the domains specified in the RoB 2.0 tool, in particular the domain pertaining to the measurement of mental health outcomes. The risk of bias assessments for the eight non-randomized studies of intervention are summarized in Fig 3. Many of the studies were assessed to be at risk of potential bias associated with residual confounding, participant selection, classification of interventions and treatment of missing data.

### Effects of activities

We present effects found in both RCTs and non-randomized studies to provide a full picture of the available evidence.

#### Exercise

Eight studies measured the effect of various forms of exercise on mental health outcomes [11–18]. While most studies examined exercise conducted in group settings, the interventions described all indicated that the activity would be possible to perform on one’s own, without an activity facilitator, and all measured outcomes at the individual level were not likely to have been obtained as part of a group experience. The studies were a mix of randomized and non-randomized trials. In one randomized trial, researchers measured state anxiety levels in 37 prisoners after the exercise group completed a six-week interval exercise training program [11]; another randomized trial measured depression, state anxiety, and trait anxiety in 60 prisoners after the exercise group underwent a 12-week exercise program [12]. In a non-randomized trial, depression and anxiety were measured in 30 prisoners with pre-existing mental health symptoms after the exercise group completed a three-month exercise program [13]; another non-randomized trial measured somatization, anxiety, social dysfunction, and severe depression in 97 prisoners, where the individuals in the exercise group exercised at least three times each week [14]. A non-randomized trial measured distress levels of 173 prisoners, which were compared to those who engaged in exercise over four weeks [15]. In another randomized trial, researchers measured symptoms of somatization, obsessive-compulsive, interpersonal sensitivity, depression, anxiety, phobic anxiety, psychoticism, paranoid ideation, and Global Severity Index (GSI) in 64 prisoners after the exercise groups completed nine months of cardiovascular plus resistance training or high intensity strength training [16]. In a non-randomized trial, depression was measured in 60 prisoners who engaged in exercise or did not exercise [17]. Another non-randomized trial measured psychological well-being in 838 prisoners and compared those scores to individuals who engaged in exercise for four weeks [18].

**Fig 4:**
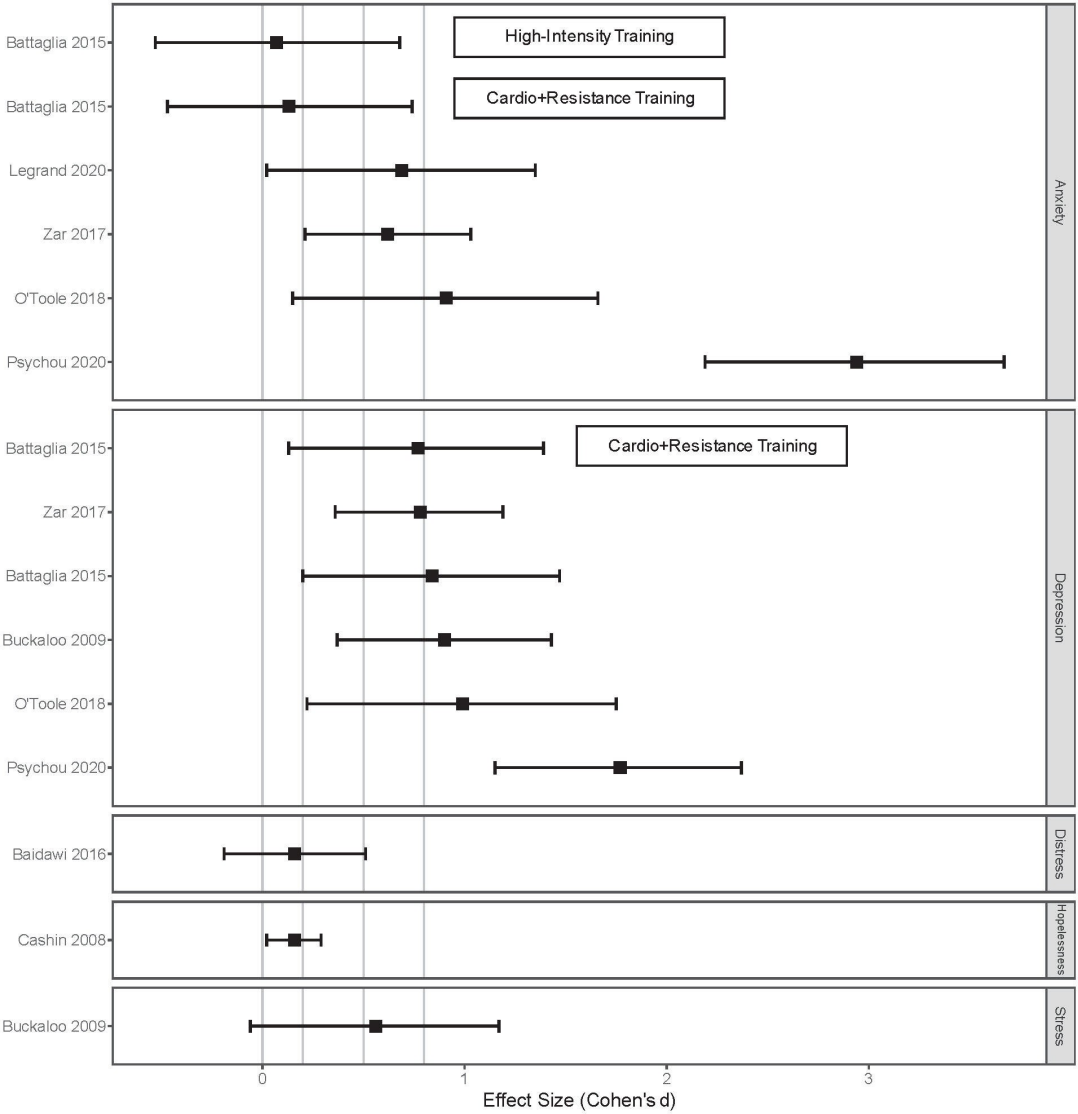
Effect size estimates for the impact of exercise on mental health.

As Fig 4 shows, the point-estimate of effect sizes obtained from exercise studies were all above zero, suggesting that exercise has beneficial effects on anxiety, depression, distress, hopelessness and stress. Due to the small sample sizes in these studies, the impact could not be measured more precisely as indicated by the wide confidence intervals, some of which even have negative lower confidence limits.

#### Yoga

Five studies measured the effect of yoga on mental health outcomes [19–23]. All of these studies examined yoga in group settings with a yoga instructor. The descriptions provided in the studies indicated that all the yoga sessions investigated would be possible to perform on one’s own, without a yoga instructor and that all measured outcomes at the individual level were not likely to have been obtained as part of a group experience. The studies were a mix of randomized and non-randomized trials. In one randomized trial, researchers measured GSI, anxiety, depression, interpersonal sensitivity, obsessive-compulsive symptoms, psychoticism, paranoid ideation, phobic anxiety, and somatization in 152 prisoners after the yoga group participated in ten weeks of hatha yoga [19] and another randomized trial measured perceived stress and psychological distress in 152 prisoners after the yoga group participated in ten weeks of hatha yoga [20]. A non-randomized trial measured anxiety, depression, and perceived stress scores from baseline compared to post-intervention and two months post-intervention in 26 forensic patients, where the experimental group completed eight weeks of mindful yoga sessions [21]. In a randomized trial, researchers measured positive affect, negative affect, perceived stress, and psychological distress in 100 prisoners, after the yoga group completed a ten-week hatha yoga program [22]. Another non-randomized trial measured depression and anxiety scores from baseline compared to 12-weeks in 21 prisoners, where the experimental group completed 12 weeks of Iyengar yoga [23].

**Fig 5:**
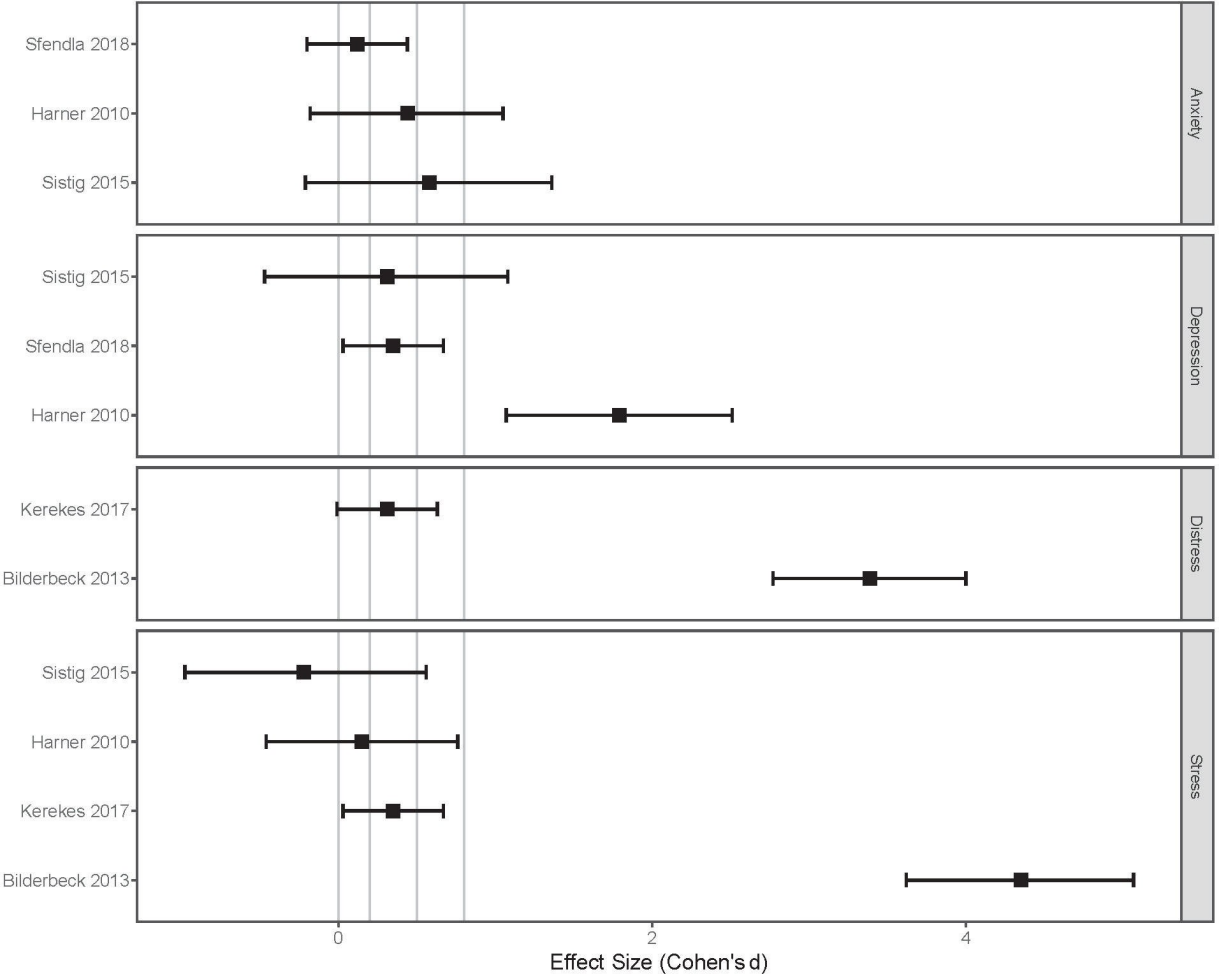
Effect size estimates for the impact of yoga on mental health.

Fig 5 shows the range of effect size estimates obtained from studies on yoga and its impact on mental health. All point-estimates, except for one study, were above zero suggesting beneficial effects, especially for alleviating depression symptoms, distress, and stress. Due to the small sample sizes in these studies and the likely high variability of the mental health outcome measures examined, the magnitude of the impact of yoga could not be measured precisely, with half of the effect size estimates having negative lower confidence intervals.

#### Relaxation

**Fig 6:**
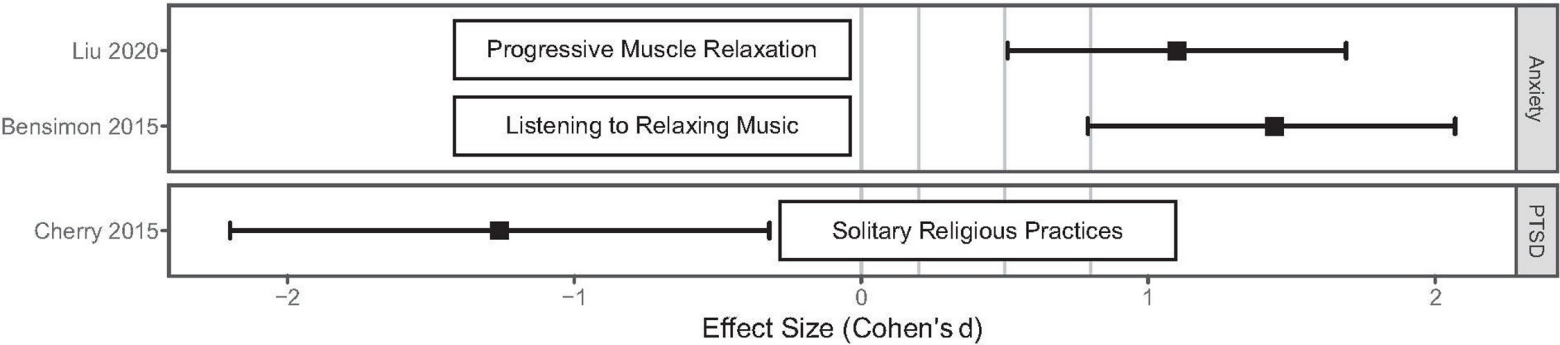
Effect size estimates for the impact of other activities on mental health.

A randomized trial investigated the effect of progressive muscle relaxation on state anxiety levels in 51 COVID-19 patients [24]. The experimental group used progressive muscle relaxation technique for 30 minutes per day for five consecutive days while the control group received routine care, with no intervention targeting state anxiety. Using the State-Trait Anxiety Inventory, the study found that mean state anxiety scores in the intervention group were lower (44.96, SD = 12.68) than the control group (57.15, SD = 9.24), with an overall effect size of 1.10 (95%CI: 0.51, 1.69) (Fig 6).

#### Music

A randomized trial investigated the effect of listening to relaxing music on state anxiety levels of 48 prisoners [25]. The experimental group was exposed to relaxing music for 45 minutes three times a day for a total of three weeks. The control group was not exposed to any music. Using the State-Trait Anxiety Inventory, the study found that immediately after the intervention, the music group reported greater reduction (from baseline) in state anxiety than the control group, with an overall effect size of 3.53 (95%CI: 2.61, 4.43). One-week post-intervention, the greater reduction (from baseline) in state anxiety in the music group was sustained, with an overall effect size of 1.44 (95%CI: 0.79, 2.07) (Fig 6).

#### Religiosity

A cross-sectional study investigated the effect of solitary religious activities (i.e. reading religious literature, watching or listening to religious programs) on PTSD of 219 coastal residents [26]. The coastal residents were from communities in south Louisiana who were affected by the 2005 Katrina and Rita hurricanes and the 2010 British Petroleum Deepwater Horizon oil spill. Using the 17-item PTSD checklist, increased odds of experiencing PTSD were reported among persons who were categorized as being medium, 2.03 (95% CI: 0.37, 11.3), and high, 9.8 (95%CI: 1.78, 53.78), in solitary religious activities. This suggests that reducing activities associated with solitary religious activities may have a beneficial impact on mental health (Fig 6).

## Discussion

This rapid review found evidence indicative of the potential role of exercise, yoga, and other activities like progressive muscles relaxation, listening to music, and religious activities on mental wellness. The eligible studies have reported mostly favourable changes or differences associated with these activities on various mental health outcomes including anxiety, depression, and psychological distress. In one study, however, a counter intuitive effect was reported suggesting a specific type of religious activity, namely solitary religious activity, is associated with higher PTSD symptoms. Overall, the methodological limitations in the eligible studies characterize the body of evidence as being at risk of bias and preclude precise estimation of the magnitude of the impact.

We posit that the risk of bias in the evidence is largely due to the use of self-report measures of mental health outcomes, participants’ partial awareness of the study purpose, outcome, and the activities they are performing, and the relatively short interval between the onset of the intervention and outcome measurement. These limitations are common in mental health research and are most of the time challenging to overcome. For example, it is often not feasible to blind participants to the study conditions if the intervention involves exercise, yoga, or relaxation. As well, participants’ adherence to or persistence in pursuing an activity is likely associated with their perception that the activity has resulted in a change in their mental well-being. Nevertheless, the use of self-reported outcomes in mental health research is acceptable if the tools have good psychometric properties for the target population, even though it can be expected that the data remains susceptible to self-reporting bias [27].

The generalizability of the evidence is high as the studies were conducted in various countries, populations, and contexts, including in very low resource settings that have a number of commonalities with what most people will experience during public health restrictions or lockdown. The findings are particularly useful and relevant to the COVID-19 pandemic because many of the activities identified can be done at home, and without the assistance of a mental health professional or trained clinician. This is important for systems with shortages of mental health professionals and for vulnerable populations who do not have access to the healthcare system due to stigma and other inequities.

It should be noted that our search did not yield studies that investigated exercise or yoga in purely individual settings. Because of this, the effect sizes reported in these studies may differ from what could be achieved if these activities were performed alone. With exercise, some studies suggest that synergistic effects can be observed if exercises are performed in group settings [28]. On the other hand, there appears to be no evidence of difference in the self-reported benefits that can be derived from performing yoga alone versus in a group setting [29].

Various pathways have been proposed to explain how the activities identified in this review might contribute to mental wellness. Most explanations postulate that effects on mental well-being are mediated through changes in physiology, brain chemistry, affect, behavior, and in perceived social support. A brief summary of the proposed pathways for each activity is presented in the next paragraphs.

**Exercise.** Exercise’s impact on mental health has mostly been attributed to its physiological and psychological affects. The anxiolytic and antidepressant properties of exercise, for example, appear to be mediated by its effects on body temperature [30], brain opioid systems [31], adrenal activity, and noradrenaline and dopamine, which play an important role in stress adaptation and mood regulation [32]. Exercise also acts as a form of distraction from stressful situations, thereby creating a sense of control, which improves overall mental wellbeing [32]. Lastly, exercise can produce short-term psychological effects such as increased feelings of vigour and self-esteem, and reduce anxiety, fatigue, and tension [33, 34].

**Relaxation. **Progressive relaxation has been shown to decrease anxiety and stress by reducing the activity of the sympathetic nervous system, which in turn increases mental relaxation [35]. The two-step process of contracting and releasing muscles activates a relaxation response, which improves the body’s overall functioning and decreases stress levels [36].

**Yoga. **The practice of yoga involves the body, breath and mind, affecting many systems simultaneously and resulting in reduced anxiety and perceived stress [37] and increased positive well-being [38]. The impact of yoga on mental well being, has been suggested to be mediated mainly by improvement in autonomic responses to stress and coping behaviours [39]. Some studies also suggest that yoga practice is linked to improved immunity and stress regulation [40] as well as better emotional regulation [41].

**Music.** Several studies highlight that listening to relaxing music reduces agitation, anxiety, anger, negative thoughts, and aggression among various populations [42]. Relaxing music is generally characterized as music with steady, slow, repetitive and flowing rhythms that are similar to a relaxed heart rate [43]. Relaxing music’s effect has been attributed to its ability to decrease sympathetic nervous system activity, state anxiety, and stress [44, 45]. Additionally, relaxing music can act as a distractor from negative stimuli, allowing listeners to focus on pleasant thoughts, which reduces stress, anxiety, and negative mood [46].

**Religious activities.** Religion and spirituality have been associated with physical and mental health including responses to stress, anxiety and depression [47–50]. Religiosity involves personal and social factors that can contribute to health and wellbeing, such as perceived social support from the religious community [50]. Perceived social support itself has been shown to correlate with reduced psychological distress, and reduced post-traumatic stress disorder [51]. Some aspects of religiosity, however, have been reported to be negatively associated with mental wellness. For example, religiosity associated with religious struggles have been found to be associated with more depression [52]. As well, some forms of religiosity that are practiced independently of a church or religious organization have been linked to higher PTSD symptoms [26, 53].

### Study limitations

Being a rapid review, this study is different in some ways from a traditional systematic review. An important difference is the expedited process we followed during full-text review and assessment of risk of bias. We restricted our search and review to articles that were published in English and that had full-texts, which may have resulted in the exclusion of potentially relevant studies. Also, our data extraction was restricted to what was provided in the full-texts, which may have affected our risk of bias assessments.

The majority of the studies included in this rapid review were conducted in prison settings, which are places characterized by acute lack of resources, lack of mobility, and lack of contact with the outside world, which resemble, in many ways the situations many people find themselves in during lockdowns. While it is likely that the activities found to promote mental wellness in prison settings may work similarly in less restrictive environments, such as during the Covid-19 pandemic, it is possible that these activities may lead to different results because of differences that may exist between the general population and persons living in prisons. As well, we note that while all the activities identified in this review can be performed on one’s own without an activity facilitator, it is possible that the effects that will be obtained will be different from those that were reported in studies conducted in group settings or with a facilitator.

Finally, our exclusive focus on home-based activities that can improve mental wellness during pandemics, post-disaster period, incarceration and scientific expeditions have excluded studies that document the impact of various activities on mental health during normal contexts. Presumably, there is a plethora of studies that examine the impact of various activities on mental health outside of pandemic situations or events that lead to decreased levels of social interaction or major disruptions in the general population. The extent to which those studies can be extrapolated to the COVID-19 pandemic is largely unknown, but it may merit further investigation. The best source of evidence for these activities are most likely to be published systematic reviews.

## Conclusions

There is some evidence that activities that can be performed at home can promote mental wellness. Physical activity such as exercise and yoga can be helpful across a broad range of symptoms of mental illness. Due to limitations in the study designs that form the evidence-base for these activities, we recommend that these activities be promoted or offered in conjunction with conventional mental health services when directed towards persons with pre-existing mental health conditions. Also, combining the activities identified in this review with standard therapies delivered online may produce better results, particularly for people experiencing moderate to severe symptoms.

Guidelines on home-based activities that have the potential to promote mental wellness are needed. These will help minimize potential adverse effects that arise when certain activities are done in excess or incorrectly (e.g. excessive or incorrect exercise routines). Guidelines would also increase the likelihood that the activities will be optimized to achieve their intended benefits. More importantly, the guidelines could include reminders about the importance of healthy use of, or exposure to social media and news [54, 55], diet, sleep hygiene recommendations (e.g. reducing or minimizing exposure to light sources before going to sleep), and sensible consumption of substances such as alcohol or caffeine.

There is a need to update this synthesis on a regular basis with results from identified studies that will be published in the next few months and beyond. Studies that use strategies and outcome measures that are less susceptible to self-reporting biases would also help increase the validity of evidence for these activities. Finally, for a medium to long term perspective, a synthesis of current systematic reviews or a meta-review would help identify a wider range of mental health promoting activities that could be rigorously tested during the ongoing and perhaps recurring pandemic periods.

## Supporting information

S1 Appendix Search strategy. (DOC)

S1 Checklist (DOC)
