# Supplementary material for: A rapid review of home-based activities that can promote mental wellness during the COVID-19 pandemic
Source: PLoS One. 2020 Dec 3;15(12):e0243125. doi: 10.1371/journal.pone.0243125 (PMC7714353; doi:10.1371/journal.pone.0243125)
Supplement: S1 Appendix — (DOC) [file pone.0243125.s001.doc]

S1 Appendix. Search StrategyOvid MEDLINE search strategy, run May 28, 2020. Details of the “covid-19” filter used in line 94 are found in the next table.

| 1 | exp Social Isolation/ | 17301 |
| --- | --- | --- |
| 2 | Quarantine/ | 2256 |
| 3 | social isolation.tw. | 6840 |
| 4 | quarantine*.tw. | 4746 |
| 5 | lockdown*.tw. | 399 |
| 6 | lock down*.tw. | 46 |
| 7 | ("stay at home" adj3 order*).tw. | 30 |
| 8 | "shelter in place".tw. | 83 |
| 9 | "shelter at home".tw. | 1 |
| 10 | forced isolation.tw. | 24 |
| 11 | (home bound or homebound).tw. | 1056 |
| 12 | (house bound or housebound).tw. | 314 |
| 13 | self-isolat*.tw. | 161 |
| 14 | confinement.tw. | 18639 |
| 15 | confined.tw. | 76536 |
| 16 | social* distan*.tw. | 1683 |
| 17 | physical* distan*.tw. | 1236 |
| 18 | or/1-17 | 122992 |
| 19 | exp exercise therapy/ | 50174 |
| 20 | exp Exercise/ | 193080 |
| 21 | motor activity/ | 96289 |
| 22 | dancing/ or exp sports/ | 183766 |
| 23 | exp Exercise Movement Techniques/ | 8120 |
| 24 | exp leisure activities/ | 234202 |
| 25 | exercis*.tw. | 290276 |
| 26 | physical* activ*.tw. | 113607 |
| 27 | danc*.tw. | 7139 |
| 28 | sport*.tw. | 73577 |
| 29 | fitness.tw. | 72374 |
| 30 | resistance training.tw. | 7476 |
| 31 | strength training.tw. | 4714 |
| 32 | walking.tw. | 70215 |
| 33 | running.tw. | 58706 |
| 34 | jogging.tw. | 1642 |
| 35 | cycling.tw. | 60317 |
| 36 | active living.tw. | 715 |
| 37 | active lifestyle.tw. | 1708 |
| 38 | yoga.tw. | 4585 |
| 39 | tai chi.tw. | 1720 |
| 40 | taiji.tw. | 85 |
| 41 | tai ji.tw. | 40 |
| 42 | qi gong.tw. | 102 |
| 43 | qigong.tw. | 700 |
| 44 | zumba.tw. | 57 |
| 45 | exergam*.tw. | 587 |
| 46 | crossfit.tw. | 148 |
| 47 | or/19-46 | 857360 |
| 48 | Mindfulness/ | 3133 |
| 49 | exp Mind-Body Therapies/ | 50165 |
| 50 | mbsr.tw. | 606 |
| 51 | mindfulness.tw. | 7080 |
| 52 | meditation.tw. | 4739 |
| 53 | relaxation.tw. | 117347 |
| 54 | or/48-53 | 169836 |
| 55 | exp Sensory Art Therapies/ | 50294 |
| 56 | art.tw. | 102023 |
| 57 | artistic.tw. | 2824 |
| 58 | music*.tw. | 20825 |
| 59 | Reading/ | 22495 |
| 60 | Cooking/ | 11909 |
| 61 | writing/ | 15226 |
| 62 | reading.tw. | 112342 |
| 63 | cooking.tw. | 14642 |
| 64 | writing.tw. | 26704 |
| 65 | or/55-64 | 341193 |
| 66 | exp Horticulture/ | 946 |
| 67 | Environment/ | 63141 |
| 68 | natur*.tw. | 1064046 |
| 69 | garden*.tw. | 12456 |
| 70 | (hike or hiking).tw. | 1022 |
| 71 | or/66-70 | 1131399 |
| 72 | exp Communications Media/ | 319358 |
| 73 | skype.tw. | 362 |
| 74 | zoom.tw. | 1580 |
| 75 | online.tw. | 112485 |
| 76 | telephon*.tw. | 59448 |
| 77 | social media.tw. | 10278 |
| 78 | social networking/ or online social networking/ | 3453 |
| 79 | podcast*.tw. | 771 |
| 80 | youtube.tw. | 1830 |
| 81 | instagram.tw. | 464 |
| 82 | Mobile Applications/ | 5696 |
| 83 | video games/ | 5333 |
| 84 | (video gam* or videogam* or online gam*).tw. | 4557 |
| 85 | social support/ | 70016 |
| 86 | self-help groups/ | 9056 |
| 87 | self help.tw. | 6436 |
| 88 | social support*.tw. | 38402 |
| 89 | social connection*.tw. | 1072 |
| 90 | social contact*.tw. | 3250 |
| 91 | or/72-90 | 582551 |
| 92 | 47 or 54 or 65 or 71 or 91 | 2900016 |
| 93 | 18 and 92 [quarantine + interventions] | 19529 |
| 94 | limit 93 to covid-19 | 247 |
| 95 | mental health.tw. | 139810 |
| 96 | psycho*.tw. | 621596 |
| 97 | emotion*.tw. | 193313 |
| 98 | distress*.tw. | 121347 |
| 99 | stress*.tw. | 821963 |
| 100 | depress*.tw. | 453284 |
| 101 | loneliness.tw. | 6034 |
| 102 | trauma.tw. | 224451 |
| 103 | anxiety.tw. | 186213 |
| 104 | anxious.tw. | 16432 |
| 105 | insomnia.tw. | 20410 |
| 106 | sleep.tw. | 162050 |
| 107 | exp mental disorders/ | 1229904 |
| 108 | Mental Health/ | 37649 |
| 109 | Resilience, Psychological/ | 5529 |
| 110 | wellness.tw. | 9489 |
| 111 | well-being.tw. | 74364 |
| 112 | wellbeing.tw. | 15371 |
| 113 | exp Adaptation, Psychological/ | 126152 |
| 114 | coping.tw. | 53058 |
| 115 | or/95-114 | 3167873 |
| 116 | 93 and 115 [quarantine + interventions + mh terms] | 6680 |
| 117 | disease outbreaks/ or epidemics/ or pandemics/ | 96551 |
| 118 | exp Disasters/ | 85190 |
| 119 | pandemic*.tw. | 30290 |
| 120 | epidemic*.tw. | 102782 |
| 121 | disaster*.tw. | 24671 |
| 122 | (SARS or SARS-CoV or MERS or MERS-CoV or Severe Acute Respiratory Syndrome or Middle East respiratory syndrome).tw. | 17674 |
| 123 | Severe Acute Respiratory Syndrome/ | 4598 |
| 124 | Expeditions/ | 1589 |
| 125 | expedition*.tw. | 3644 |
| 126 | exp Prisons/ or exp Prisoners/ | 23144 |
| 127 | prison*.tw. | 16203 |
| 128 | incarcerat*.tw. | 11445 |
| 129 | (detention or detaine*).tw. | 4559 |
| 130 | or/117-123 | 295063 |
| 131 | 116 and 130 | 181 |
| 132 | 94 or 131 [search limited to pandemic/disaster terms] | 358 |
| 133 | or/124-129 | 43366 |
| 134 | 130 or 133 | 337131 |
| 135 | 116 and 134 | 261 |
| 136 | 94 or 135 [including also expeditions and incarceration] | 438 |
| 137 | limit 136 to (english language and yr="2000 -Current") | 381 |

Details of covid-19 Ovid MEDLINE filter, as found at:

<https://ospguides.ovid.com/OSPguides/medline.htm>

| 1 | exp Coronavirus/ |
| --- | --- |
| 2 | exp Coronavirus Infections/ |
| 3 | (coronavirus* or corona virus* or OC43 or NL63 or 229E or HKU1 or HCoV* or ncov* or covid* or sars-cov* or sarscov* or Sars-coronavirus* or Severe Acute Respiratory Syndrome Coronavirus*).mp. |
| 4 | (or/1-3) and ((20191* or 202*).dp. or 20190101:20301231.(ep).) [this set is the sensitive/broad part of the search] |
| 5 | 4 not (SARS or SARS-CoV or MERS or MERS-CoV or Middle East respiratory syndrome or camel* or dromedar* or equine or or coronary or coronal or covidence* or covidien or influenza virus or HIV or bovine or calves or TGEV or feline or porcine or BCoV or PED or PEDV or PDCoV or FIPV or FCoV or SADS-CoV or canine or CCov or zoonotic or avian influenza or H1N1 or H5N1 or H5N6 or IBV or murine corona*).mp. [line 5 removes noise in the search results] |
| 6 | ((pneumonia or covid* or coronavirus* or corona virus* or ncov* or 2019-ncov or sars*).mp. or exp pneumonia/) and Wuhan.mp. |
| 7 | 2019-ncov or ncov19 or ncov-19 or 2019-novel CoV or sars-cov2 or sars-cov-2 or sarscov2 or sarscov-2 or Sars-coronavirus2 or Sars-coronavirus-2 or SARS-like coronavirus* or sarscov-2 or Sars-coronavirus2 or Sars-coronavirus-2 or SARS-like coronavirus* or coronavirus-19 or covid19 or covid-19 or covid 2019 or ((novel or new or nouveau) adj2 (CoV or nCoV or covid or coronavirus-19 or covid19 or covid-19 or covid 2019 or ((novel or new or nouveau) adj2 (CoV or nCoV or covid or coronavirus* or corona virus or Pandemi*2)) or ((covid or covid19 or covid-19) and pandemic*2) or (coronavirus* and pneumonia)).mp. |
| 8 | COVID-19.rx,px,ox. or severe acute respiratory syndrome coronavirus 2.os. |
| 9 | ("32240632" or "32236488" or "32268021" or "32267941" or "32169616" or "32267649" or "32267499" or "32267344" or "32248853" or "32246156" or "32243118" or "32240583" or "32237674" or "32234725" or "32267344" or "32248853" or "32246156" or "32243118" or "32240583" or "32237674" or "32234725" or "32173381" or "32227595" or "32185863" or "32221979" or "32213260" or "32205350" or "32202721" or "32197097" or "32196032" or "32188729" or "32176889" or "32088947" or "32277065" or "32273472" or "32273444" or "32145185" or "31917786" or "32267384" or "32265186" or "32253187" or "32265567" or "32231286" or "32105468" or "32179788" or "32152361" or "32152148" or "32140676" or "32053580" or "32029604" or "32127714" or "32047315" or "32020111" or "32267950" or "32249952" or "32172715").ui. [Articles not captured by this search when created in April 2020, pending further indexing by NLM] |
| 10 | or/6-9 [Lines 5 to 8 are specific to Covid-19] |
| 11 | 5 or 10 |
| 12 | 11 and 20191201:20301231.(dt). |
| 13 | remove duplicates from 12 |
